# Current State and Challenges of Local Production of Vaccines in Nigeria

**DOI:** 10.1002/puh2.70006

**Published:** 2024-10-10

**Authors:** Obi Peter Adigwe, Godspower Onavbavba, Olajide Joseph Adebola, Anthony Ayeke, Saheed Ekundayo Sanyaolu, Kenneth Anene Agu

**Affiliations:** ^1^ National Institute for Pharmaceutical Research and Development Abuja Federal Capital Territory Nigeria; ^2^ Home Plus Medicare Services Ltd. Abuja Federal Capital Territory Nigeria; ^3^ European Union Delegation to the Federal Republic of Nigeria and ECOWAS Abuja Federal Capital Territory Nigeria; ^4^ Howard University Office of International Programs Howard University Global Initiative Nigeria Abuja Federal Capital Territory Nigeria

**Keywords:** Africa, cold chain, healthcare, immunisation, infectious diseases, manufacturing, public health

## Abstract

**Background:**

Vaccination protects the population against infectious diseases and reduces their transmissibility. Potentials exist for local production of vaccines in Nigeria, as a means of addressing public health needs. However, challenges exist in certain critical aspects which limit development in this area. This study aimed at evaluating the challenges of local vaccines’ manufacturing in Nigeria from the perspectives of relevant stakeholders.

**Methods:**

This was a cross‐sectional study. A structured questionnaire was used for data collection. The data obtained from the study were analysed descriptively.

**Results:**

More than half of the study participants (55.5%) agreed that significant gaps exist with respect to access to vaccines in Nigeria. Only about one‐quarter of the respondents (25.8%) were of the view that relevant legislative frameworks exist to support government funding in the area of vaccine production. One‐third of the participants (32.3%) expressed confidence in the availability of trained human resources for vaccine production. Close to two‐thirds of the respondents (61.7%) expressed dissatisfaction regarding the current funding for vaccine research and development, and a similar proportion (65.2%) were of the opinion that a lack of local manufacturing capacity contributed to the sub‐optimal access to vaccines. Moreover, two‐thirds (62.3%) disagreed that Nigeria was prepared for future pandemics.

**Conclusion:**

Ill‐suited policies, sub‐optimal infrastructure, and inadequate research and development funding, are some factors which the study identified as contributory to the lack of access to vaccines in Nigeria. There is a need to improve incentives, infrastructural development and build human resource capacity for vaccine research and development to enhance local production in Nigeria.

## Graphical abstract

• The study revealed that inadequate funding for research and development activities has contributed to the lack of access to vaccines in Nigeria.

• The findings from this study demonstrated the need to improve incentives, infrastructural development, as well as build human resource capacity for vaccine research and development.

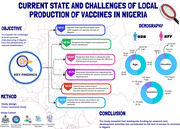

## 1 Introduction

Vaccination is a preventive measure that protects the population against certain infectious diseases and reduces their transmissibility. Mass vaccination strategies have controlled several infectious diseases such as smallpox, polio and measles [1]. The process involves introducing antigenic material from a pathogen into the body, and this stimulates immune responses such as antibodies, memory cells and other factors, without causing the actual disease [2, 3]. The benefits of vaccination transcend individual protection and contribute to the concept of herd immunity, a phenomenon whereby the vaccination of a significant proportion of the population confers immunity to vulnerable individuals who have not been vaccinated [4]. In addition to promoting good public health, vaccines prevent the financial burden of treating diseases and save families from high medical costs, thereby maintaining their financial stability [5].

According to the World Health Organization, at least 30 million children in Africa under the age of 5 years get infected with vaccine‐preventable diseases annually. Over half a million of these children die from these conditions due to a lack of access to immunisation services in the continent [6]. Nigeria, Africa's most populated country, faces significant challenges in meeting its immunisation needs. Available evidence suggests that only a third of children aged 12–23 months received complete routine vaccination in 2018, and up to 19% received none [7].

Despite the lessons from COVID‐19 pandemic, local production of vaccines still remains a critical challenge in Nigeria and several developing countries. This has resulted in the importation of all vaccines utilised in the Nigerian setting [8]. Africa accounts for only 3% of global pharmaceutical production, and depending on the location, between 70% and 90% of pharmaceuticals utilised in the continent are imported [9]. Nigeria's current reliance on imported vaccines has several downsides, including supply chain delays and disruptions. Others include the high cost of importation, and the inability to safeguard the public during disease outbreaks [10]. As at the peak of COVID‐19 pandemic, the demand for vaccines by the global community delayed the supply of this important public health intervention tool to Nigeria [11, 12]. Local manufacturing of vaccines can therefore strengthen the response to public health emergencies and enhance national healthcare delivery.

The importance of vaccine research in enhancing production capabilities cannot be overemphasised. Research and development are critical at every step of vaccine production [13]. Furthermore, vaccine research allows the development of vaccinations tailored to the needs of specific demographic groups and ensures consistent quality in production processes [14]. Research activities also enable a swift response to emerging infectious diseases by exploring genetic techniques to expedite the development of vaccines against newly identified pathogens.

Several studies have evaluated the feasibility of vaccine production in several low‐ and middle‐income countries [10, 15–18]. However, there is a paucity of information on relevant research with a robust assessment of challenges militating against local manufacturing of vaccines in Nigeria and other African countries. Therefore, this study aimed at assessing the views of relevant stakeholders in the vaccine research, development and manufacturing value chain, with a view to identifying challenges as well as developing contextual strategies to expedite local production.

## 2 Methods

### 2.1 Study Design, Setting and Population

The research followed a cross‐sectional study approach, deployed among participants who attended a webinar organised to promote local production of vaccines in Nigeria. The participants included healthcare professionals; pharmaceutical manufacturers; academics and researchers; regulatory affairs specialists; members of professional bodies; and other relevant stakeholders in the vaccine value chain.

### 2.2 Study Variables, Instrument and Data Collection

A convenience sampling strategy was adopted to include everyone who participated in the webinar for data collection. The data were collected using a structured questionnaire that was designed following a comprehensive review of literature [19, 20, 21, 22, 23]. Face and content validations of the questionnaire were carried out by a panel of experienced researchers. The pilot testing of the questionnaire was undertaken by administering it to a cohort of 20 randomly selected participants. The feedback from the testing resulted in no significant change to the questionnaire. The data collection instrument had two sections that included socio‐demographic characteristics and items to assess participants’ views using a five‐point Likert scale. Data were collected between June and September 2023.

An electronic consent form was sent via email to the participants. Only those who completed and returned the consent form received the questionnaire created using Google Forms. A reminder was also sent to the participants to increase the response rate. The information provided were anonymised, and ethical principles of ensuring confidentiality were maintained.

### 2.3 Data Analysis

Data collected were coded into the Statistical Package for Social Sciences (SPSS) version 25. Univariate analysis was carried out to yield descriptive statistics, and data were presented in frequencies as well as percentages. Responses received from the five‐point Likert scale were aggregated to arrive at Agree, Neutral and Disagree.

## 3 Results

A total of 505 respondents participated in this study. Female respondents (54.9%) consituted the greater proportion of the study participants. One‐third of the participants (35.8%) were between the ages of 31 and 40 years. Three‐quarters of the study sample (76.9%) were first‐degree holders, whereas PhD holders represented the least proportion (2.8%). Further details on socio‐demographic characteristics are provided in Table 1.

**TABLE 1:** Socio‐demographic characteristics of participants who attended a webinar on local production of vaccines in Nigeria in June 2023 (*n* = 505).

Variable	Frequency (%)
Sex	
Male	228 (45.1)
Female	277 (54.9)
Age (years)	
18–30	238 (47.1)
31–40	181 (35.8)
41–50	49 (9.7)
51–60	35 (6.9)
Above 60	2 (0.4)
Highest educational qualification	
Diploma	36 (7.2)
First degree	386 (76.9)
Master's degree	66 (13.1)
Doctorate	14 (2.8)
Years of experience in current work	
Less than 5 years	245 (48.9)
5–10 years	159 (31.7)
11–15 years	38 (7.6)
Above 15 years	59 (11.8)
Sector of practice	
Government sector	252 (50.0)
Private sector	226 (44.8)
Development agency	9 (1.8)
Others	17 (3.4)
Area of employment	
Pharmaceutical manufacturing	64 (14.3)
Non‐pharmaceutical manufacturing	391 (85.7)

*Note:* Missing data were excluded from the percentages presented in Table 1.

**FIGURE 1:**
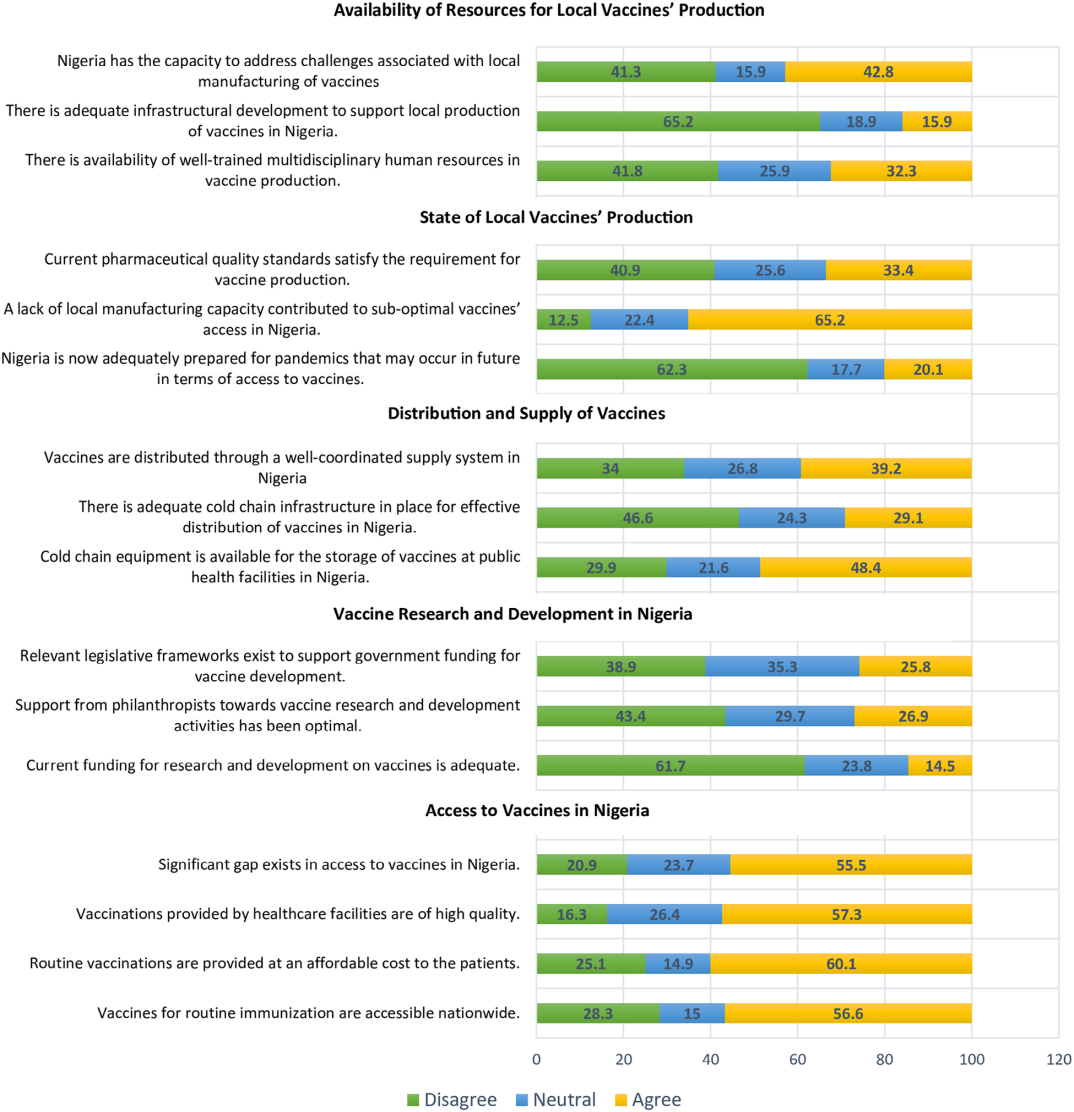
Current state and challenges of local production of vaccines.

Figure 1 captures participants' perception regarding availability of resources for local production of vaccines; state of production; distribution and supply chain; research and development activities; and access to this important public health tool. From the findings presented in Figure 1, the respondents agreed that significant gaps exist in the area of access to vaccines in Nigeria. Only a quarter of the participants agreed that relevant legislative frameworks exist to support government funding in the area of vaccines. The participants were of the view that the current funding for research and development activities was inadequate.

Additionally, a significant proportion of the sample disagreed that support from philanthropists towards vaccine research and development activities was optimal. Participants were of the view that Nigeria was not prepared for future pandemics in terms of access to vaccines. Furthermore, the respondents were of the opinion that there was a lack of adequate infrastructural development to support local production of vaccines in Nigeria.

## 4 Discussion

This study provides insights into challenges militating against local manufacturing of vaccines in Nigeria. Findings revealed that significant gaps exist in terms of access to vaccines in the country, and this corroborates previous findings [24]. Over the years, support from the Global Alliance for Vaccines and Immunisation (GAVI) and other donor organisations contributed towards enhancing routine immunisation coverage in Nigeria [25]. Despite this support, adequate immunisation coverage has not been achieved, as evidence suggests that about a quarter of children aged 2 years and below in this setting remain unvaccinated, thereby leaving them exposed to vaccine‐preventable diseases [26]. With the planned transition from GAVI, there is a need to develop a robust framework for the local manufacturing of vaccines to ensure contextual access to this essential public health tool.

In the context of vaccine research and development, the participants indicated that there was a lack of relevant legislative frameworks to support government funding in this area. This is a critical challenge which has been linked with sub‐optimal access to vaccines, despite the high demand for this public health tool to address infectious disease burden in the country. Although measures to support access to vaccines have increased in Nigeria since the COVID‐19 pandemic [27], there exist significant gaps in the aspects of funding. Participants also indicated that government funding for vaccine research and development was inadequate, suggesting the need for a comprehensive and contextual strategy to address issues in this area. Other studies reported insufficient funding as an impediment to conducting research in Nigeria [28, 29, 30]. Additionally, more than a third of the respondents disagreed that the support from philanthropists regarding vaccine research and development was optimal. This underscores the need to engage with development partners and philanthropic organisations to improve relevant support. According to global health reports, mobilisation of funds from bilateral agencies and philanthropies constitutes a major source of financing for vaccines research and development [30]. This is evident within the Asia‐Pacific region, where the Bill and Melinda Gates Foundation has funded human papillomavirus (HPV) vaccine research and development at the Serum Institute of India as well as measles and rubella vaccine development at Bio Farma in Indonesia [31]. Considerable mobilisation of development partners and  philanthropic support for research and development in Nigeria can significantly promote innovation and local manufacturing in the vaccine landscape.

Findings from this study revealed inadequacy in cold chain infrastructure, which is critical for vaccines’ storage and distribution. This is similar to earlier results from a study that was undertaken in Oyo state, Nigeria [32]. Cold chain is a critical aspect of the vaccine distribution system as it ensures that vaccines are stored and transported at the appropriate temperature to maintain their potency and efficacy. Improving vaccine distribution requires investment in infrastructures which include cold chain equipment, a stable power supply and an effective logistic system among others [23, 33, 34]. These measures will significantly address the disruptions in cold chain storage, consequently stimulating vaccine development in the country. Regarding the availability of resources for local vaccine production in Nigeria, a considerable proportion of the study sample expressed disagreement with the adequacy of the current infrastructure. This finding is in line with previous studies [35, 36, 37]. According to the World Health Organization, the challenges with infrastructural deficits for vaccine development in Nigeria may border on the lack of or outdated nature of the relevant facilities, which in turn, stifle or totaly prevent necessary vaccine related activities [38]. This underscores the urgent need to promote access to new investments in this area as a means of addressing the gaps in infrastructural development towards vaccine production in the country.

Furthermore, only about a third of the study sample agreed that well‐trained multidisciplinary human resources are available for vaccine production in Nigeria, suggesting a lack of capacity in this area. This challenge has been similarly identified across other countries within the sub‐Saharan African region [39], corroborating reports that the region constitutes only 3% of the global health workforce [40]. Appropriately skilled personnel with applied knowledge and access to modern vaccine technologies and infrastructure are imperative to enhance local vaccine research and development and production. To therefore increase the vaccine development capacity in Nigeria, there is a need to allocate significant resources towards providing comprehensive training for relevant professionals across all aspects of the value chain, including research, manufacturing and regulatory processes. This measure has proven effective across other vaccine‐producing countries [41] and can serve as an effective priority pathway to expanding sustainable vaccine manufacturing in settings such as Nigeria.

Alongside the deficits of a skilled workforce for vaccine development as presented in this study, the majority of the sample were of the view that Nigeria is not prepared to handle pandemic situations that may occur in future. This was expressed due to the current state, where all vaccines used in the country are imported. Sub‐optimal vaccine access was attributed to the absence of local manufacturing capacity. As of August 2023, only about a third of the Nigerian population had two doses of the COVID‐19 vaccine [42]. Although vaccine hesitancy played a role in this situation [43], a significant contributing factor is the inadequate availability of vaccines for domestic utilisation within Nigeria [44]. Furthermore, only about a third of the participants expressed confidence in the suitability of the current pharmaceutical quality standards for vaccine production. The field of vaccine manufacturing is continuously evolving with advances in science and technology; hence, quality improvement initiatives are key to the commencement of local vaccine production in Nigeria. The findings from this study revealed the need for more responsive policies and incentives within the vaccine manufacturing value chain. This approach would assure the intensification of relevant efforts aimed at achieving a supportive ecosystem.

Whilst this study revealed some novel findings, it is important to comprehensively explore critical areas such as market access, sustainability partnerships, and technology transfer. Further studies are therefore required to build on this work's findings and how they interplay with multidisciplinary complementary factors. Future policies and practices can leverage these emergent strategies to develop ecosystems that improve access to vaccines and enable the achievement of Universal Health Coverage.

## 5 Conclusion

This study provides novel insights into the challenges related to local production of vaccines in Nigeria. Deficiencies were observed in several areas, including a lack of adequate funding for relevant activities which consequently contributed to the current state of widespread sub‐optimality in vaccine research, development and produc.

The findings from this study shed light on shortfalls within the national vaccine value chain and provide perspectives into key priority areas that demand immediate and strategic intervention. There is a need for government and policymakers to develop contextual strategies to support funding of research activities as well as improve relevant infrastructure and equipment critical for the local production of vaccines in Nigeria.

## Author Contributions

**Obi Peter Adigwe:** conceptualisation, supervision. **Godspower Onavbavba** and **Saheed Ekundayo Sanyaolu:** data curation, writing–original draft. **Obi Peter Adigwe**, **Godspower Onavbavba**, **Anthony Ayeke**, **Saheed Ekundayo Sanyaolu** and **Kenneth Anene Agu:** formal analysis. **Obi Peter Adigwe**, **Godspower Onavbavba**, **Olajide Joseph Adebola** and **Kenneth Anene Agu:** investigation. **Obi Peter Adigwe**, **Godspower Onavbavba**, **Olajide Joseph Adebola**, **Anthony Ayeke**, **Saheed Ekundayo Sanyaolu** and **Kenneth Anene Agu:** methodology. **Godspower Onavbavba:** project administration. **Anthony Ayeke:** resources. **Obi Peter Adigwe**, **Godspower Onavbavba**, **Olajide Joseph Adebola**, **Anthony Ayeke** and **Kenneth Anene Agu:** validation. **Olajide Joseph Adebola** and **Anthony Ayeke:** visualisation. **Obi Peter Adigwe**, **Olajide Joseph Adebola**, **Anthony Ayeke** and **Kenneth Anene Agu:** writing–review and editing.

## Ethics Statement

The study was conducted in accordance with the Declaration of Helsinki. Ethical approval was obtained from the National Institute for Pharmaceutical Research and Development Health Research Ethics Committee prior to the commencement of data collection  (approval number: NIPRD‐HREC 25/04/2023‐26).

## Consent

Participation in this study was voluntary, as informed consent was obtained from the participants prior to the administration of questionnaires.

## Conflicts of Interest

The authors declare no conflicts of interest.

## Supporting information

Supporting Information

## Data Availability

The datasets used and/or analysed during this study are available from the corresponding author on request.
