# Supplementary material for: Current State and Challenges of Local Production of Vaccines in Nigeria
Source: Public Health Chall. 2024 Oct 10;3(4):e70006. doi: 10.1002/puh2.70006 (PMC12039692; doi:10.1002/puh2.70006)
Supplement: Supplementary file 1 — Supporting Information [file PUH2-3-e70006-s001.docx]

**Questionnaire**

**Current State and Challenges of Local Production of Vaccines in Nigeria: An Assessment of Views of Stakeholders in the Value Chain**

**Introduction**

Local production of vaccines can help sustain access to high quality products, improve access to healthcare services, and also prevent the shortage of supply as experienced for COVID-19 pandemic. This study aims at better understanding the issues underpinning the current state of local manufacturing of vaccines in Nigeria. The information you provide will be anonymised and treated confidentially.

**Demographic Data**

1. **Gender**

| Male | Female |
| --- | --- |

1. **Age (years)**

| 18-30 | 31-40 | 41-50 | 51-60 | Above 60 |
| --- | --- | --- | --- | --- |

1. **Highest Educational Qualification**

| Diploma | First degree | Master’s degree | Doctorate |
| --- | --- | --- | --- |

1. **Years of Experience in Your Current Work**

| >5years | 5-10years | 11-15years | Above 15years |
| --- | --- | --- | --- |

1. **Sector of Practice**

| Government Sector | Private Sector | Development Agency | Others, please specify ………………………………. |
| --- | --- | --- | --- |

1. **Area of Employment**

| Pharmaceutical Manufacturing | Non-Pharmaceutical Manufacturing |
| --- | --- |

**Current State and Challenges of Local Production of Vaccines in Nigeria**

1. **State of Vaccine Provision in Nigeria**

| **SN** | **Statements** | **Strongly Disagree** | **Disagree** | **Neutral** | **Agree** | **Strongly Agree** |
| --- | --- | --- | --- | --- | --- | --- |
|  | Vaccines for routine immunization are accessible nationwide. |  |  |  |  |  |
|  | Routine vaccinations are provided at an affordable cost to the patients. |  |  |  |  |  |
|  | Vaccinations provided by healthcare facilities are of high quality. |  |  |  |  |  |
|  | Significant gap exists in access to vaccines in Nigeria. |  |  |  |  |  |

1. **Vaccine Research and Development in Nigeria**

| **SN** | **Statements** | **Strongly Disagree** | **Disagree** | **Neutral** | **Agree** | **Strongly Agree** |
| --- | --- | --- | --- | --- | --- | --- |
|  | Current funding for research and development on vaccines is adequate. |  |  |  |  |  |
|  | Support from philanthropists towards vaccine research and development activities has been optimal. |  |  |  |  |  |
|  | Relevant legislative frameworks exist to support government funding for vaccine development. |  |  |  |  |  |
|  |  |  |  |  |  |  |

1. **Distribution and Supply of Vaccines**

| **SN** | **Statements** | **Strongly Disagree** | **Disagree** | **Neutral** | **Agree** | **Strongly Agree** |
| --- | --- | --- | --- | --- | --- | --- |
|  | Cold chain equipment is available for the storage of vaccines at public health facilities in Nigeria. |  |  |  |  |  |
|  | There is adequate cold chain infrastructure in place for effective distribution of vaccines in Nigeria. |  |  |  |  |  |
|  | Vaccines are distributed through a well-coordinated supply system in Nigeria |  |  |  |  |  |

1. **State of Local Vaccine Production**

| **SN** | **Statements** | **Strongly Disagree** | **Disagree** | **Neutral** | **Agree** | **Strongly Agree** |
| --- | --- | --- | --- | --- | --- | --- |
|  | Nigeria is now adequately prepared for pandemics that may occur in future in terms of access to vaccines. |  |  |  |  |  |
|  | A lack of local manufacturing capacity contributed to sub-optimal vaccines’ access in Nigeria. |  |  |  |  |  |
|  | Current pharmaceutical quality standards satisfy the requirement for vaccine production. |  |  |  |  |  |

1. **Availability of Resources for Local Vaccine Production**

| **SN** | **Statements** | **Strongly Disagree** | **Disagree** | **Neutral** | **Agree** | **Strongly Agree** |
| --- | --- | --- | --- | --- | --- | --- |
|  | There is availability of well-trained multidisciplinary human resources in vaccine production. |  |  |  |  |  |
|  | There is adequate infrastructural development to support local production of vaccines in Nigeria. |  |  |  |  |  |
|  |  |  |  |  |  |  |
|  | Nigeria has the capacity to address challenges associated with local manufacturing of vaccines |  |  |  |  |  |

1. **Support for Local Vaccine Manufacturing in Nigeria**

| **SN** | **Statements** | **Strongly Disagree** | **Disagree** | **Neutral** | **Agree** | **Strongly Agree** |
| --- | --- | --- | --- | --- | --- | --- |
|  | Government policies support local vaccine manufacturing. |  |  |  |  |  |
|  | Relevant incentives exist that support the manufacturing of vaccines in Nigeria. |  |  |  |  |  |
|  | International support for local production of vaccines in Nigeria is adequate |  |  |  |  |  |
